# Impact of omega-3 fatty acid oral therapy on healing of chronic venous leg ulcers in older adults: Study protocol for a randomized controlled single-center trial

**DOI:** 10.1186/s13063-019-3970-7

**Published:** 2020-01-16

**Authors:** Jodi C. McDaniel, Jamie Rausch, Alai Tan

**Affiliations:** College of Nursing, The Ohio State University, 372 Newton Hall, 1585 Neil Avenue, Columbus, OH 43210-1289 USA

**Keywords:** Venous leg ulcers, Fatty acids, Fish Oil, Inflammation, Polymorphonuclear leukocytes

## Abstract

**Background:**

This trial addresses the global problem of chronic venous leg ulcers (CVLUs), wounds that cause significant infirmity for an estimated 9.7 million people annually, mainly older adults with comorbidities. Advanced therapies are needed because standard topical therapies are often ineffective or yield only short-term wound healing. Thus, we are testing a new oral therapy containing the bioactive elements of fish oil, eicosapentaenoic acid (EPA) and docosahexaenoic acid (DHA), for targeting and reducing the high numbers of activated polymorphonuclear leukocytes (PMN) in wound microenvironments that keep CVLUs “trapped” in a chronic inflammatory state.

**Methods:**

This double-blind RCT will include 248 eligible adults ≥ 55 years of age with CVLUs receiving standard care at a large Midwest outpatient wound clinic. Participants are randomized to two groups: 12 weeks of daily oral therapy with EPA + DHA (1.87 g/day of EPA + 1.0 g/day of DHA) or daily oral therapy with placebo. At 0, 4, 8, and 12 weeks, across the two groups, we are pursuing three specific aims: Aim 1. Compare levels of EPA + DHA-derived lipid mediators, and inflammatory cytokines in blood and wound fluid; Subaim 1a. Compare inflammatory cytokine gene expression by PMNs in blood; Aim 2. Compare PMN activation in blood and wound fluid, and PMN-derived protease levels in wound fluid; Aim 3. Compare reduction in wound area, controlling for factors known to impact healing, and determine relationships with lipid mediators, cytokines, and PMN activation. Subaim 3a. Compare frequency of CVLU recurrence and levels of study variables in blood between the randomly assigned two subgroups (continuing EPA + DHA therapy versus placebo therapy beyond week 12) within the EPA + DHA group with healed CVLUs after 3 months of therapy. Subaim 3b. Compare symptoms of pain at all time points and quality of life at first and last time points across the two groups and two subgroups.

**Discussion:**

This trial will provide new evidence about the effectiveness of EPA + DHA oral therapy to target and reduce excessive PMN activation systemically and locally in patients with CVLUs. If effective, this therapy may facilitate healing and thus be a new adjunct treatment for CVLUs in the aging population.

**Trial registration:**

ClinicalTrials.gov, NCT03576989; Registered on 13 June 2018.

## Background

Chronic venous leg ulcers (CVLUs) are open skin lesions of the lower legs arising in areas affected by venous hypertension that have been present for at least 6 weeks [1]. These common conditions affect 1% of the adult population and 3.6% of people older than 65 years, and the incidence of CVLUs is rising because they mainly occur in older adults with comorbidities, a growing population [2]. These recurring, disabling wounds cause substantial infirmity [3]. Quality of life (QoL) declines due to related pain, reduced mobility, and protracted treatments that cost payers in the United States alone up to $15 billion per year [4].

Multilayer compression bandaging is the gold standard therapy for CVLUs to reduce venous hypertension in the lower extremities implicated in the pathobiology of CVLUs [5]. However, even with compression therapy, the median time to complete healing of uncomplicated CVLUs is reported to be 12 weeks; older (> 6 months) and larger ulcers (> 20 cm^2^) require significantly more time to heal [6]. Other data indicate that nearly 60% of CVLUs remain unhealed after 12 weeks of compression therapy and usually recur [7, 8]. Thus, new therapies to augment compression bandaging and other standard topical therapies are needed to improve CVLU outcomes and help prevent recurrence [9].

The pathobiology of CVLUs also involves high numbers of activated polymorphonuclear leukocytes (PMNs) in venous circulation and ulcer microenvironments that promote unremitting inflammation and delay healing [10–12]. Although PMNs (e.g., neutrophils) are essential for a short time in the first stage of wound healing to fight off potential pathogens [13], sustained high levels of PMNs typically found in CVLU microenvironments are detrimental to healing. Sustained high levels of PMNs lead to excessive amounts of PMN-derived proteases such as human neutrophil elastase that ultimately destroy newly formed tissue and degrade growth factors, receptors, and the extracellular matrix that are essential for healing [10, 11, 14, 15]. Excessive, prolonged levels of PMNs keep ulcers “trapped” in a chronic inflammatory state. Further, high levels of activated PMNs in venous circulation are involved in CVLU onset [9, 16, 17]. As such, reducing excessive PMN activation systemically and locally is expected to resolve inflammation, advance ulcers through subsequent stages of healing, and deter recurrence.

Studies involving cell cultures and animal models of inflammation show that the bioactive omega-3 polyunsaturated fatty acids (PUFAs) contained in fish oil, eiocosapentaenoic acid (EPA) and docosahexaenoic acid (DHA), are metabolized to eicosanoids and other lipid mediators that inhibit PMN migration to inflamed sites and reduce cell synthesis and secretion of pro-inflammatory cytokines involved in recruiting and activating PMNs [18–21]. Some human studies report lower chemotactic responsiveness of PMNs from whole blood after just 4 weeks of EPA + DHA oral therapy in healthy subjects [22, 23]. After ingestion and rapid incorporation into cell membranes, EPA + DHA are metabolized to lipid mediators that are weak chemoattractants for PMNs. Increasing EPA + DHA levels has also been shown to reduce levels of arachidonic acid (an omega-6 PUFA) that is metabolized to lipid mediators that upregulate pro-inflammatory cytokine synthesis [24–26]. Moreover, there is evidence that EPA + DHA decrease the actual gene expression of pro-inflammatory cytokines in cells by blocking nuclear factor kappa B activity [27, 28]. Specific to wounds, our early pilot work demonstrated that EPA + DHA oral therapy can push an EPA + DHA lipid mediator profile in acute human wound microenvironments that is associated with declining levels of myeloperoxidase, a biomarker of PMN activation [29]. In addition, data from our more recent study showed that EPA + DHA reduces PMN activation in blood and wound fluid [25], and reduces levels of proinflammatory cytokines in venous circulation of patients with CVLUs [30] after 4 and 8 weeks of therapy. While these prior studies have made notable scientific contributions, there is a critical need to expand understanding of the physiological processes responsible for the link between EPA + DHA oral therapy, reduced PMN activation, and CVLU pathobiology.

Therefore, this double-blind randomized controlled trial (RCT) will include 248 eligible adults with CVLUs, randomized to two groups: 12 weeks of daily oral therapy with EPA + DHA (1.87 g/day of EPA + 1.0 g/day of DHA) or daily oral therapy with placebo. At 0, 4, 8, and 12 weeks, across the two groups, we are pursuing three specific aims: Aim 1. Compare levels of EPA + DHA-derived lipid mediators, and inflammatory cytokines in blood and wound fluid; Subaim 1a. Compare inflammatory cytokine gene expression by PMNs in blood; Aim 2. Compare PMN activation in blood and wound fluid, and PMN-derived protease levels in wound fluid; Aim 3. Compare reduction in wound area, controlling for factors known to impact healing, and determine relationships with lipid mediators, cytokines, and PMN activation. Subaim 3a. Compare frequency of CVLU recurrence and levels of study variables in blood between randomly assigned two subgroups (continuing EPA + DHA therapy versus placebo therapy beyond week 12) within the EPA + DHA group with healed CVLUs after 3 months of therapy. Subaim 3b. Compare symptoms of pain at all time points and quality of life at first and last time points across the two groups and two subgroups. The findings are expected to close a significant gap in knowledge about the potential for EPA + DHA oral therapy, as an adjunct to compression therapy, to facilitate healing and prevent recurrence of CVLUs. Our organizing clinical hypothesis is that increasing EPA + DHA intake with oral supplementation will expedite CVLU healing and prevent recurrence by raising levels of EPA + DHA-derived lipid mediators and lowering levels of pro-inflammatory cytokines that will reduce PMN activation systemically and in the wound microenvironment. This research is expected to make a positive impact on individuals, families, and caregivers affected by CVLUs because if EPA + DHA therapy reduces chronic excessive PMN activation, improves healing outcomes and helps prevent recurrence, the significant emotional and financial burdens associated with the pain, social isolation, lost productivity, and protracted health care visits will be reduced.

## Methods

### Design and ethical considerations

**Fig. 1:**
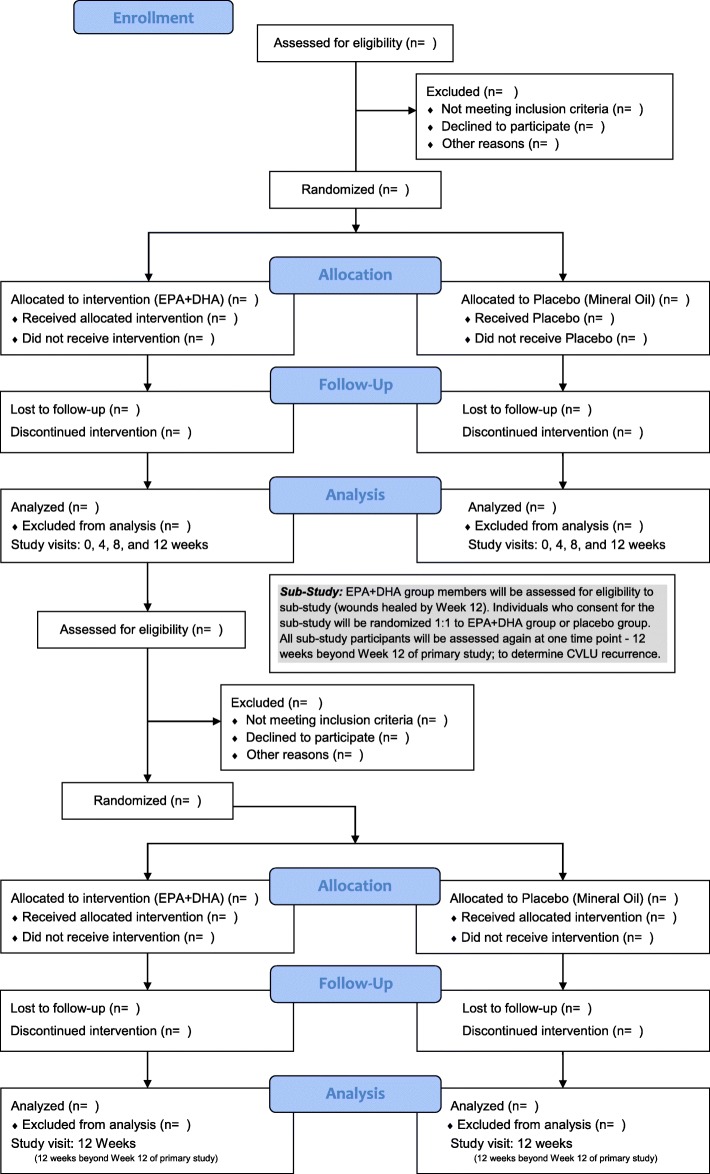
Study flow chart

This ongoing trial uses a prospective two-group, double-blind, randomized, repeated-measures, placebo-controlled design. Participants are studied at 0, 4, 8, and 12 weeks after eligibility screening, written informed consent, and enrollment to the study. A subset of participants in the EPA + DHA group with healed CVLUs by 12 weeks will be studied for an additional 3 months so that we can determine the frequency of CVLU recurrence. The trial was approved by the local institutional review board of the participating medical center (The Ohio State University [OSU] Wexner Medical Center – July 20, 2018). Figure 1 and Table 1 show the study flow chart and the schedule, respectively.

**Table 1:** Study schedule

Time point	Pre-Visit	Visit 1 (Week 0)	Visit 2 (Week 4)	Visit 3 (Week 8)	Visit 4 (Week 12)	Visit 5* (3 mos. Post Week 12)
Enrollment						
Screening	X					
Informed consent		X				
Allocation		X				
Interventions						
Treatment (EPA + DHA)		X	X	X	X	X
Placebo (mineral oil)		X	X	X	X	
Assessments						
Wound treatments	X	X	X	X	X	X
Adherence			X	X	X	X
Biological measures						
Blood and wound fluid		X	X	X	X	X
Fatty acids		X	X	X	X	X
n-3, n-6 lipid mediators		X	X	X	X	X
Cytokines		X	X	X	X	X
PMN activation		X	X	X	X	X
Proteases (wound fluid only)		X	X	X	X	X
Wound area (if wound is present)		X	X	X	X	X
Body mass index		X	X	X	X	X
CEAP		X	X	X	X	X
Revised VCSS (includes pain score)		X	X	X	X	X
Interview/self-report						
Sociodemographic Questionnaire		X				
Perceived Stress Scale		X	X	X	X	X
Health history		X				
Food frequency Questionnaire		X			X	X
VEINES-QoL/Sym Questionnaire		X			X	X

Legend: X = Collected at the given time point. n-3 = Omega 3; n-6 = Omega 6; PMN = polymorphonuclear leukocytes; CEAP = Clinical, Etiologic, Anatomic, and Pathophysiologic classification; VCSS = Venous Clinical Severity Score; VEINES-QoL/Sym = VEnous Insufficiency Epidemiological and Economic Study – Quality of Life/Symptoms. *All assessments at visit 5 are for participants initially randomized to the treatment group, have healed wounds by visit 4, and are consented for the additional study. At visit 4, these participants will be re-randomized to the treatment group or placebo group and have one more study visit, 3 months following visit 4

### Setting

We are recruiting participants over a 53-month period from a pool of patients diagnosed with CVLUs who are receiving treatment with standard single- or multi-layer compression bandaging at the OSU Comprehensive Wound Center (CWC). The study visits occur at the OSU Clinical Research Center, located on the medical center’s main campus.

### Eligibility criteria

Patients are eligible for the study if they are women and men ≥ 55 years of age (1) with a CVLU between the ankle and knee that has been present for at least 4 weeks, but not longer than 12 months, are prescribed compression therapy with 1–4 layer bandaging; (2) with an ankle brachial pressure index (ABPI) between 0.7 and 1.2; (3) with a target wound area of 2–60 cm^2^, (4) who can read and understand English or Spanish, and (5) who can provide consent. Patients are included after full, understandable, and neutral explanation by the project manager or principal investigator (PI) and after giving written informed consent. Patients are excluded when they report a fish allergy or when they receive a treatment or have a condition known to seriously impair normal wound healing (e.g., corticosteroids, selective COX-2 inhibitors, non-steroidal anti-inflammatory drugs >2x/week (exception: aspirin 81 mg/day), chemotherapy, autoimmune diseases, diabetes if HbA1c > 12%, or a venous leg ulcer complicated by cellulitis, exposed tendon or bone).

### Sample size

Sample size was determined to have sufficient power to detect intervention effect on wound healing, the optimal outcome of the study. Using mixed-effects linear modeling for repeated measures, a total of 248 participants (124 per group) are needed to have 80% power to detect an effect size of 0.4 for between-group comparisons in percent wound area reduction. We assumed (1) a first-order autoregressive covariance structure among the three post-intervention repeated measures, (2) a within-subject correlation of 0.7, and (3) an attrition rate of 20% in the sample size calculation. These assumptions are reasonable based on data from our pilot study [25]. The effect size of 0.4 is corresponding to an average of 16% between-group difference in percent wound area reduction with a common standard deviation of 0.4. Our power analysis also applied Bonferroni method to adjust for three comparisons of primary interest (4-week vs. baseline, 8-week vs. baseline, and 12-week vs. baseline). We used the same approach to conduct power analysis for other outcomes. Our sample size will have over 90% power to detect intervention effects on levels of lipid mediators and inflammatory cytokines and PMN activation, which had medium to large effect sizes based on our pilot study [25, 29]. A two-sided significance level of 0.05 was used for all power analyses. Based on OSU CWC data, we will be able to recruit the required number of people (*n* = 248) in the 53-month recruitment period, averaging 4–5 per month.

#### Recruitment and retention plans

We are recruiting participants from a pool of patients diagnosed with CVLUs scheduled to begin standard care (1–4 layer compression bandage) at the OSU CWC. Because of the known effect of aging on wound healing, the age distribution of CVLU patients at the CWC and participants in our R21 study [25], our target recruitment is 168 patients aged 55–69 years and 80 patients aged ≥ 70 years. The project manager identifies eligible patients by reviewing CWC clinical records. After CWC clinicians present a letter that introduces our team to the potential participants, the project manager provides study details and confirms eligibility of patients interested in participating in the study. After enrollment, the project manager contacts participants every 2 weeks to answer questions and remind them of study visits. The project manager or PI obtains informed written consent from participants at visit 1. Participants are also asked for permission for the research team to share relevant data with people from the university taking part in the research or from regulatory authorities, where relevant. Participants receive $250 after completing the 12-week study; subgroup participants receive an additional $150 after completing visit 5. There is no anticipated harm for trial participation and thus no provisions for post-trial care or compensation.

### Randomization and intervention

After written informed consent is obtained, patients eligible for the study are randomly assigned to either the treatment group or the placebo group by the project manager using the stratified permuted block randomization scheme generated by the study statistician. Precisely, we divide participants in each age stratum (55–69 vs. 70+ years) into permuted blocks of varying block sizes (4 or 8 people/block). Those in each block will be randomly allocated with a ratio of 1:1 to either of the two study arms. Those in each arm will consume softgels of allocated therapy (EPA + DHA or placebo) daily for 12 weeks. Stratified permuted block randomization ensures balanced randomization across age groups and over time. The varied block sizes were chosen to prevent predictability in treatment allocation. The study participants, clinicians, and researchers are blinded as to treatment.

#### EPA + DHA softgels

Three opaque EPA + DHA softgels (J.R. Carlson Laboratories, Inc.; Arlington Heights, IL, USA) will provide a total daily intake of 1.87 g EPA and 1.0 g DHA. This dose/ratio is proposed because EPA has relatively stronger anti-inflammatory properties than DHA [31], and a similar dose/ratio significantly raised EPA + DHA plasma levels and reduced n-6:n-3 ratios in humans after 4 weeks in our pilot work [25]. The Federal Drug Administration reports that ≤ 3.0 g/d of EPA + DHA is safe for public use [32]. At weeks 0, 4, and 8, participants are given one bottle with the ensuing 1-month supply of EPA + DHA or placebo softgels. A 3-month supply of supplements will be supplied at week 12 (visit 4) for those participants that qualify and enroll in the sub-study.

#### Placebo softgels

Three opaque placebo softgels will provide a total daily dose of 2.5 ml of mineral oil (well below therapeutic dose of 10 ml for constipation). Mineral oil is chemically inert, and on ingestion 98% remains unabsorbed in feces. We have used the same placebo dose in prior studies [25, 33]. The EPA + DHA and placebo softgels will be the same in appearance, lemon-flavored and packaged in like bottles (J.R. Carlson Laboratories, Inc.; Arlington Heights, IL, USA).

### Clinical management

#### Monitoring clinic wound treatments

Standard of care for uncomplicated CVLUs in the CWC involves a silver impregnated dressing under a 1–4 layer compression bandage. However, circumstances (e.g., infection) may arise that require a clinician to alter treatment*.* The project manager conducts weekly wound treatment monitoring checks using a standardized checklist to review participants’ clinic records for changes in wound treatments. Any treatment change (e.g.,oral antibiotic) is recorded. We will control for treatment-related variables by considering them covariates as appropriate.

#### Monitoring adherence

At each study visit, the subsequent month’s supply of softgels (one bottle) and written and verbal directions for consuming/storing softgels are given to each participant. At subsequent study visits, the bottles are collected. The number of softgels remaining in bottles are counted and logged for each participant. The project manager contacts participants every 2 weeks to review instructions and promote adherence. Adherence is verified by measuring EPA + DHA levels in blood plasma at each study visit to assess changes over time.

### Data and laboratory measurements

All data are anonymized and collected using electronic report forms by investigators or trained research personnel at each study visit who are blinded to the study group assignments. Adverse events are recorded (e.g., gastrointestinal upset). Blood and wound fluid samples are collected at each study visit for use in laboratory tests: quantification of fatty acids, lipid mediators, PMN activation, cytokine levels, and cytokine gene expression.

### Wound fluid collection

Wound fluid is collected from unhealed ulcers at each study time point using a standard wound fluid collection protocol [25, 29]. The fluid is collected by the PI experienced in the protocol or CRC nurses trained by the PI. Briefly, after CVLUs are washed with sterile water, a transparent occlusive film (Opsite, Smith & Nephew, UK) is applied over the wound and the leg is placed in a dependent position for approximately 1–1½ hours. While slowly removing the occlusive film and rinsing the wound with 1 ml of sterile saline, the fluid is collected using a 26G × 0.5″ angiocatheter attached to a 3-ml syringe (Terumo Medical, Somerset, NJ, USA). The fluid is transferred into plain collection tubes and analyzed immediately to determine PMN activation or frozen and stored at − 80^°^ C until further analysis.

### Primary outcome measures

The primary outcome measure with respect to the effectiveness of EPA + DHA oral therapy in the treatment of CVLUs is time to complete wound healing. We define complete wound healing as reepithelialization of the total wound surface. The PI who is blinded to treatment assesses this outcome. The area of unhealed ulcers is quantified at each time point in cm^2^ using a single digital camera photogrammetry system [34]. We calculate percent reduction in ulcer area at weeks 4, 8, and 12 compared to week 0 for each participant and averages for each group. Larger percentages indicate greater healing. The second primary outcome measure is PMN activation in blood and CVLU microenvironments. The determination of PMN activation is accomplished using flow cytometry. Additional measures to assess PMN activation in CVLU fluid involve quantifying reactive oxygen species production during “respiratory burst” by PMNs and two PMN-specific protease biomarkers (matrix metalloproteinase-8, human neutrophil elastase). We also measure free fatty acids in plasma and erythrocyte membranes, lipid mediators in plasma and CVLU fluid, and a panel of pro- and anti-inflammatory cytokines in plasma and CVLU fluid. Finally, inflammatory cytokine gene expression in PMNs (neutrophils and monocytes) is measured.

### Secondary outcome measures

Pain is assessed at all time points using the revised Venous Clinical Severity Score (VCSS). This revised VCSS is a valid and reliable instrument to measure severity of venous disease, pain specific to chronic venous disease of the legs, and response to treatment over time that can be compared across studies [35]. Quality of life is measured at weeks 0 and 12 using the Venous Insufficiency Epidemiological and Economic Study-Quality of Life/Symptoms **(**VEINES-QOL/Sym) questionnaire, based on the Short Form – 36 (SF-36) questionnaire [36]. VEINES-QOL/Sym is a standardized, 26-item, patient-reported questionnaire to assess the severity and frequency of venous insufficiency symptoms.

CVLU recurrence will be assessed in two subgroups derived from the EPA + DHA group. Unblinding will occur at week 12. If participants in the EPA + DHA group have healed CVLUs by week 12, they will be asked to consider participating in a continuing study to determine the preliminary efficacy of continuing EPA + DHA therapy in preventing ulcer recurrence. If participants agree to participate, they will be randomly assigned 1:1 to either of two subgroups: subgroup 1 will continue EPA + DHA therapy for three more months, while subgroup 2 will receive placebo therapy. The PI and participants will be blinded to treatment. Three months later, participants in the subgroups will return for a final study visit (visit 5).

### Covariates

To address potential confound of variables that may impact inflammation or wound healing [37], we are collecting data on age, sex, tobacco use, comorbidities, perceived stress, and prescribed/over-the-counter medications. We are also collecting data on dietary intake of nutrients that may affect healing (e.g., vitamin C and protein) using a food frequency questionnaire [38]. All instruments being used to measure tobacco use, perceived stress, and dietary intake have been well vetted so data can be compared across studies. We are also determining if wound debridement or adjunct therapies were used between study visits and noting the type of dressing under compression bandaging.

### Statistical analysis

Congruent with the RCT design of the study, we will conduct an intent-to-treat analysis. We will first use descriptive statistics to check data distribution, identify outliers, guide appropriate data transformation if needed, and summarize sample characteristics. Two-sample *t* tests and Chi-square statistics will be used to check the balance of baseline measures between two study arms. Mixed-effects linear modeling for repeated measures will be used to test the intervention effect on each outcome measure (PMN activation, lipid mediators, inflammatory cytokines, inflammatory cytokine gene expression, percent reduction in wound area, and pain and QoL). In each model, we will include fixed-effects of treatment (EPA + DHA vs. control), time, and treatment by time interaction, adjusting for possible covariates (e.g., lipid-lowering medications) and data dependancy from repeated measures. From the mixed-effects modeling, we will derive the between-group contrast (EPA + DHA vs. control) estimates of change from baseline in outcome variable at each follow-up time point. Multiple comparison adjustment (e.g., Bonferroni adjustment) will be applied to these between-group contrasts to avoid inflated type I error. Logistic regression modeling will be used to analyze wound recurrence. Again, we will estimate between-group difference in the risks of wound recurrence, adjusting for potential covariates and multiple comparison. Lastly, we will use longitudinal mediation modeling [39] to test hypothesized mediating pathways. A significant mediation pathway is indicated if both paths from X (e.g., treatment) to M (mediator, e.g., pro-inflammatory cytokine) and from M to Y (e.g., PMN activation) are statistically significant. Adequate model fit of a mediation model will be indicated with a non-significant Chi-square statistic, comparative fit index, and Tucker & Lewis Index ≥ 0.9, and a root mean square error of appromixation of ≤ 0.08 [40]. The effect of biological sex and its interaction with treatment will also be examined in the regression models. Missing Data. We expect missing data due to non-adherence, loss of follow-up, or lack of data due to healed wounds. We will carefully examine the extent and pattern of missing data. We will use an indicator variable to flag missing data due to healed wounds and incoporate the variable in the missing pattern analysis. Mixed-effects modeling allows for missing at random [41]. If missing completely at random (MCAR) exists, multiple imputation will be used in order to use the optimal amount of information in our analysis. For missing not at random, pattern-mixture modeling will be used [42]. We will also conduct sensitivity analysis to examine the robustness of study findings from different methods. The trial is considered “minimal risk” by the local IRB and therefore no interim analysis or formal plans for stopping the trial are planned. In terms of data and safety monitoring, a Safety Officer (SO) has been selected and approved by the funding agency in consultations with the PI. The SO has no financial, scientific, or other conflict of interest with the trial. The SO meets with the PI twice annually to review study progress, data quality, and participants’ safety.

#### Protocol amendments

The PI will notify the sponsor/funder of this project of any changes to the protocol and will submit the changes for review to the local IRB. The PI will notify the clinical research center (study site) of any protocol changes and will update the protocol in the clinical trial registry.

## Discussion

Chronic venous leg ulcers are wounds that cause substantial morbidity, disability, hospitalization, and even mortality among older adults [3, 4]. New therapies are needed because prevalence rates for CVLUs are rising and standard topical therapies are often ineffective [7, 8, 43]. The high levels and expression of PMN-derived proteases in fluid and tissues biopsied from CVLUs [44–46, 47], and the significantly higher levels of PMN-derived proteases found in chronic wounds compared with healing wounds [48–50] indicate that sustained high levels of PMN-derived proteases are factors in healing delays. There is a body of evidence that the omega-3 fatty acids contained in fish oil (EPA + DHA) mitigate PMN activation [18, 25, 27, 50, 51]. The main goals of this study are to determine the effects of combination therapy with oral EPA + DHA supplementation and standard compression bandaging on PMN activation, CVLU healing, and CVLU recurrence. The findings from this trial are expected to lead to a new low-risk adjunct therapy to target and reduce the excessive PMN activation involved in CVLU pathobiology, stimulate healing, and lessen the negative impact CVLUs have on QoL.

In this study, we have chosen wound area in cm^2^ and PMN activation in systemic circulation and in the wound microenvironment as the primary endpoints. Persistent PMN activation and the resulting high levels of PMN-derived proteases in wound microenvironments are well correlated with slow healing, while lower levels are associated with faster healing [50]. Therefore, this primary outcome measurement is a logical approach to evaluate the effectiveness of oral EPA + DHA therapy to target a specific biological process linked to CVLUs. In addition, we will assess the potential mechanisms of action of the intervention, (EPA + DHA-derived lipid mediators and pro- and anti-inflammatory cytokines) and the secondary endpoints of pain, QoL, and CVLU recurrence.

The omega-3 PUFAs EPA + DHA are metabolized to lipid mediators such as prostaglandin E3 that are weak chemoattractants for PMNs. They also produce the resolvin species that actively slows migration of PMNs to inflammatory sites, triggering inflammation resolution [18, 19]. In vivo experiments in a murine model of peritonitis after zymosam challenge indicated that DHA-derived resolvin D3 reduces PMN transmigration by 45% [52]. Other studies have shown that EPA + DHA-derived lipid mediators slow chemotaxis of PMNs by reducing expression or antagonism of receptors for chemoattractants [53] and by down-regulating gene expression of pro-inflammatory cytokines that signal PMN activation [27]. The studies by these groups were well designed and executed, but there is a need to determine the extent that EPA + DHA therapy increases EPA + DHA-derived lipid mediators and reduces pro-inflammatory cytokine synthesis in more in vivo studies of humans with conditions such as CVLUs linked to excessive PMN activation, versus cell and animal models of inflammation. There is also a need to determine effects of EPA + DHA therapy on omega-6 PUFA-derived lipid mediators. The n-3 and n-6 PUFA families are competitively metabolized and n-6 PUFAs such as arachidonic acid generate lipid mediators that are strong chemoattractants for PMNs (e.g., leukotriene B4) and signal gene expression of pro-inflammatory cytokines in cells [20, 54].

At the end of this trial, we expect to know the extent that EPA + DHA therapy and the proposed mediating factors reduce PMN activation, expedite CVLU healing, and prevent recurrence. We expect to know more about the mechanisms of action of EPA + DHA therapy and its subsequent impact on ulcer pain and QoL. This knowledge will propel us toward our long-term goal *–* to help prevent or facilitate healing of CVLUs in aging and thereby improve QoL. After this study, next steps may include (1) a pragmatic trial to test EPA + DHA therapy in the everyday clinical setting to maximize generalizability, assess cost effectiveness, and determine the feasibility of using a point-of-care test [55] that can measure protease levels in CVLU fluid within minutes, (2) determining the extent that EPA + DHA therapy prevents CVLUs in people with known venous disease, and (3) determining the extent that genetic factors affect EPA + DHA metabolism. We predict that EPA + DHA oral therapy added to standard topical care will greatly improve CVLU healing outcomes and as such, reduce the substantial emotional and financial burdens caused by these serious wounds in aging (Additional file 1).

## Trial status

Protocol version #3, April 24, 2019. Registered at Clinicaltrials.gov, identifier: NCT03576989. Currently recruiting, with nine participants enrolled. Trial start date was April 15, 2019. Anticipated recruitment end date is March 15, 2023.

## Supplementary information

**Additional file 1.** SPIRIT 2013 Checklist: Recommended items to address in a clinical trial protocol and related documents*.

## Data Availability

Information and datasets gathered as a result of this trial will be available from the corresponding author upon reasonable request. Results and findings of the study will be released through publications in scientific literature and conference presentations.

## Abbreviations

ABPI: Ankle brachial pressure index
CRC: Clinical research center
CVLU: Chronic venous leg ulcer
CWC: Comprehensive wound center
DHA: Docosahexaenoic acid
EPA: Eicosapentaenoic acid
HbA1c: Hemoglobin A1c
MCAR: Missing completely at random
n-3: Omega 3
n-6: Omega 6
OSU: Ohio State University
PI: Primary investigator
PMN: Polymorphonuclear leukocytes
PUFA: Polyunsaturated fatty acids
QoL: Quality of life
SO: Safety officer
VCSS: Venous Clinical Severity Score
VEINES-QOL/Sym: Venous Insufficiency Epidemiological and Economic Study-Quality of Life/Symptoms
